# Stem Cell-Derived Extracellular Vesicles for Treating Joint Injury and Osteoarthritis

**DOI:** 10.3390/nano9020261

**Published:** 2019-02-14

**Authors:** Jiao Jiao Li, Elham Hosseini-Beheshti, Georges E. Grau, Hala Zreiqat, Christopher B. Little

**Affiliations:** 1 Raymond Purves Bone and Joint Research Laboratories, Institute of Bone and Joint Research, Kolling Institute, Northern Sydney Local Health District, Faculty of Medicine and Health, University of Sydney, St Leonards, NSW 2065, Australia; christopher.little@sydney.edu.au; 2 Biomaterials and Tissue Engineering Research Unit, School of Aerospace, Mechanical and Mechatronic Engineering, University of Sydney, Sydney, NSW 2006, Australia; hala.zreiqat@sydney.edu.au; 3 Australian Research Council Training Centre for Innovative BioEngineering, Sydney, NSW 2006, Australia; 4 Vascular Immunology Unit, Discipline of Pathology, Faculty of Medicine and Health, University of Sydney, Sydney, NSW 2006, Australia; elham.beheshti@sydney.edu.au (E.H.-B.); georges.grau@sydney.edu.au (G.E.G.)

**Keywords:** extracellular vesicles, exosomes, microvesicles, stem cells, mesenchymal stem cells, joint injury, osteoarthritis, joint degeneration, joint inflammation, regenerative medicine

## Abstract

Extracellular vesicles (EVs) are nanoscale particles secreted by almost all cell types to facilitate intercellular communication. Stem cell-derived EVs theoretically have the same biological functions as stem cells, but offer the advantages of small size, low immunogenicity, and removal of issues such as low cell survival and unpredictable long-term behaviour associated with direct cell transplantation. They have been an area of intense interest in regenerative medicine, due to the potential to harness their anti-inflammatory and pro-regenerative effects to induce healing in a wide variety of tissues. However, the potential of using stem cell-derived EVs for treating joint injury and osteoarthritis has not yet been extensively explored. The pathogenesis of osteoarthritis, with or without prior joint injury, is not well understood, and there is a longstanding unmet clinical need to develop new treatments that provide a therapeutic effect in preventing or stopping joint degeneration, rather than merely relieving the symptoms of the disease. This review summarises the current evidence relating to stem cell-derived EVs in joint injury and osteoarthritis, providing a concise discussion of their characteristics, advantages, therapeutic effects, limitations and outlook in this exciting new area.

## 1. Introduction

Extracellular vesicles (EVs), including exosomes, microvesicles and apoptotic bodies, are nanoscale intercellular messengers secreted by cells to deliver biological signals. EVs are becoming a new area of investigation in regenerative medicine as potential therapeutics for controlling inflammation, repairing injury and enhancing regeneration in numerous diseases [1]. However, despite the known roles of EVs in a range of physiological functions and pathological conditions, their potential in promoting joint repair and slowing degeneration has not been thoroughly investigated [2]. Faced with the global burden of osteoarthritis as the fastest growing major health condition and the leading cause of disability in the ageing population [3], research into developing EVs as therapeutic products may fulfill critical unmet clinical needs in osteoarthritis management, and potentially provide a curative solution. This review will provide a concise summary on current research into stem cell-derived EVs for the prevention of degeneration and the promotion of regeneration within the context of joint injury and osteoarthritis, and will discuss their general characteristics, therapeutic effects, limitations and outlook in relation to these novel applications.

## 2. The Burden of Osteoarthritis and Currently Available Treatments

Osteoarthritis is a leading cause of disability, affecting over 15% of the global population [1]. The lifetime risk of developing symptomatic osteoarthritis is estimated to be 25% in the hip and 45% in the knee, respectively, and the risk increases for individuals with a history of joint injury [4,5]. The disease involves inflammation, cartilage degradation and structural changes in the affected joint, resulting in severe pain and functional disability that significantly impair an individual’s ability to perform the activities of daily living. There is currently no curative treatment for this disease.

For joint injuries or cartilage damage that have not yet progressed to degenerative changes, current clinical treatments focus on attempting to relieve the symptoms of injury, such as pain and swelling, and are associated with numerous drawbacks. Reparative techniques such as microfracture often lead to the formation of fibrocartilage that lacks clinical durability [6], while restorative techniques such as osteochondral grafts are limited by the availability of donor tissue and morbidity at the donor site [7]. Cell-based strategies exemplified by autologous chondrocyte implantation (ACI) are time-consuming, have very limited shelf-life, and face problems of graft delamination and insufficient cartilage regeneration [8]. In addition, all of these existing treatments have relatively short-term effects, and do not specifically prevent the later development of osteoarthritis.

For joints that show degenerative changes or where symptomatic osteoarthritis is present, a range of non-operative treatments are used clinically, but these largely only manage the symptoms until progressive joint degeneration becomes so severe that a total joint replacement must be performed. Non-operative treatments can be divided into non-pharmacological and pharmacological treatments. Non-pharmacological treatments mainly focus on patient access to information and education, weight loss, and controlled exercise programs, but there is debate surrounding their limited effects on early symptoms and structural disease modification [9]. Pharmacological treatments mainly involve analgesics and non-steroidal anti-inflammatory drugs (NSAIDs) to reduce pain. However, due to the high incidence of co-morbidities in osteoarthritis patients, pharmacological treatments are associated with inappropriate polypharmacy and an increased risk of dangerous side effects [10]. 

Intra-articular injections of corticosteroids or hyaluronic acid can be indicated for patients whose symptoms cannot be controlled with other non-operative treatments. These injections have yielded variable results, with some evidence supporting short-term (1–6 months) effects on pain relief and functional improvement [11]. However, corticosteroid injections may lead to further joint degradation, and the analgesic effects of hyaluronic acid are controversial [11]. Both also necessitate repeated injections at least once every 6 months for sustained effects. 

Total joint replacement is the ultimate procedure for osteoarthritis patients who have failed non-operative management therapies. Although joint replacement procedures remove the diseased joint and replace its functions with an implant, these procedures are associated with increased risks of surgical complications and limited implant lifetime of approximately 20 years [12]. It is obvious that all of the currently available treatments for osteoarthritis have numerous limitations, and more importantly, have little effect in slowing disease progression. An alternative therapy that can address these challenges is urgently needed. 

## 3. Role of Stem Cells in Treating Joint Injury and Osteoarthritis

Mesenchymal stem cells (MSCs) are the most prominent cell type explored for their therapeutic potential in treating joint injury and osteoarthritis, most commonly those isolated from the bone marrow [13], adipose tissue [14], and synovium [15]. The use of MSCs in clinical trials for a wide spectrum of disease indications has increased exponentially in recent years [16], some of which investigated their efficacy in promoting tissue repair or reducing symptoms in cartilage injury or osteoarthritis [17]. For instance, intra-articular injections of MSCs to treat knee osteoarthritis in Phase I and II clinical trials were shown to be safe and well tolerated, with short-term (up to 5 years) results demonstrating improvements in pain and joint function, as well as cartilage quality [18,19,20].

The initial motivation behind MSC transplantation to treat joint diseases arose from their multilineage differentiation potential to become mesenchymal cell types (such as bone, cartilage and fat) that are relevant for musculoskeletal repair, as well as their ability to be administered through allogeneic therapy without eliciting an immune response [21]. However, many studies later reported that despite functional improvement or even joint tissue regeneration following MSC transplantation into diseased joints, their engraftment and subsequent differentiation into relevant cell types were rare [22]. Following on from these observations, the paracrine effects of MSCs came into the spotlight, and it is now generally accepted that MSCs primarily exert their therapeutic effects through the secretion of trophic factors to reduce inflammation and enhance repair [23]. The secretory functions of MSCs are likely responsible for their well-documented beneficial effects on applications relevant to joint repair, including: 1) anti-inflammatory properties, with evidence of being able to downregulate the secretion of inflammatory signals by cells in osteoarthritic cartilage, including interleukin (IL)-1β, IL-6, IL-8, matrix metalloproteinase (MMP)-1, and MMP-13 [11,24], and 2) trophic properties, characterised by the secretion of molecules that induce cell proliferation, reduce scar tissue formation, and stimulate endogenous cartilage repair, including transforming growth factor (TGF)-β, insulin-like growth factor (IGF)-1, basic fibroblast growth factor (bFGF), vascular endothelial growth factor (VEGF), and epithelial growth factor (EGF) [24,25].

Despite the multitude of benefits offered by MSCs in treating joint injury and osteoarthritis, there are also many challenges associated with direct cell transplantation, due to the limited survival of cells following injection, inability to accurately predict long-term cell behaviour and cell-cell interactions, and difficulties in maintaining a sufficiently large bank of cells to allow off-the-shelf therapy [26]. Donor variations constitute another significant challenge, as MSCs from aged or diseased donors are known to have reduced proliferation and functionality [27]. Furthermore, the need for extensive ex vivo expansion of MSCs prior to transplantation often leads to induction of senescence, loss of proliferative potential, and reduced differentiation capacity particularly beyond 10–20 population doublings [27]. Other challenges are associated with the intrinsic biological properties of MSCs. Due to their genetic conditioning to undergo calcification following chondrogenic induction as part of the natural process of endochondral ossification, difficulties have been experienced in maintaining a stable cartilage phenotype in differentiated MSCs and preventing them from progressing towards osteogenesis [28], which is a major issue to consider in cartilage repair applications. MSCs also have unique ‘environmentally-responsive’ properties and undergo distinct changes in behaviour in response to microenvironmental cues [25]. While this is a useful property that is often exploited for regenerative medicine applications, it may negatively influence the response of MSCs in a diseased joint environment. For instance, human adipose tissue-derived MSCs have been shown to change to a pro-inflammatory secretome when exposed to tumor necrosis factor (TNF) and contribute to intensifying the inflammatory response [29]. For these reasons, a novel strategy has recently emerged whereby the secretory products of MSCs, such as MSC-derived EV, are tested in experimental models of joint injury and osteoarthritis rather than the cells themselves.

## 4. Role of Stem Cell-Derived Extracellular Vesicles in Treating Joint Injury and Osteoarthritis

Stem cell-derived EVs have similar biological functions as stem cells, but offer significant advantages such as their small size, low immunogenicity, and removal of the common issues associated with direct cell injection. Initially described as ‘platelet dust’ in the 1960s [30], EVs have attracted intense research interest over the last few decades, and they are now widely recognised as powerful intercellular messengers with critical roles in mediating pathological processes, maintaining tissue homeostasis, and regulating physiological functions [31,32]. EVs have already been explored as novel therapeutics in a range of applications, including anti-tumour therapy, pathogen vaccination, immunomodulatory and regenerative therapies, and drug delivery [33]. Nevertheless, research into using stem cell-derived EV as a therapeutic tool for treating joint injury and osteoarthritis has only just begun to emerge in the last 3–5 years, and no specific reviews have yet been published on this topic. Despite the scarcity of studies comprising this young and exciting area of EV research, the available evidence is pointing to a strong potential of stem cell-derived EVs in promoting joint repair and providing protection from degeneration following joint injury.

### 4.1. General Characteristics of Extracellular Vesicles

Extracellular vesicles are a collective term for heterogeneous small, double-layered lipid membrane vesicles typically in the size range of 30–2000 nm, which act as carriers for a variety of biologically active signalling molecules, including RNA species (mRNA, microRNA), proteins, enzymes, lipids and DNA fragments [34]. They are produced by almost all cell types, including immune cells (T-cells, B-cells, dendritic cells, neutrophils, platelets), connective tissue cells (epithelial cells, fibroblasts), specialised cells (endothelial cells, neuronal cells), pathological cells (cancerous cells), and stem cells (MSCs), and are found in various biological fluids, including blood, urine, saliva, and synovial fluid [35]. Once released, EVs can act locally or be transported through the circulation to distant sites, where they achieve intercellular communication with recipient cells by reproducing the effects of their cells of origin.

**Figure 1:**
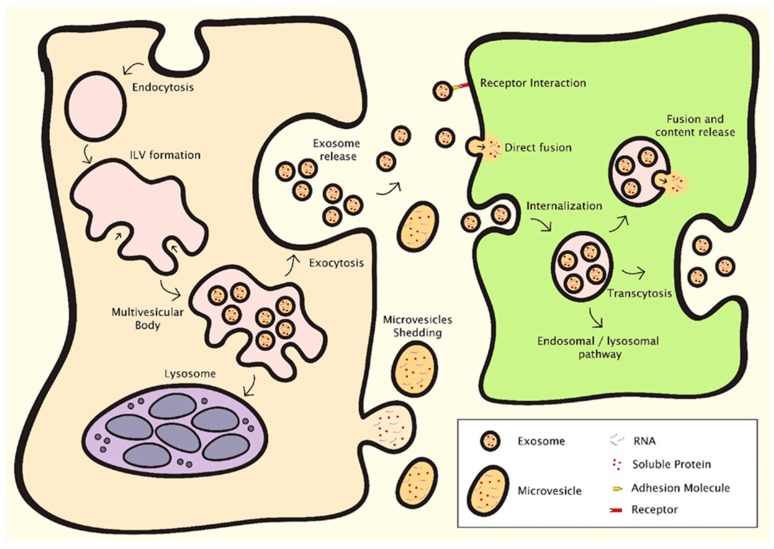
Mechanisms of EV formation and intercellular communication [38]. Exosomes and microvesicles are the EV types relevant in regenerative medicine, which contain nucleic acids, proteins and lipids. Exosomes are derived from the endosomal compartment through endosomal membrane invagination to form multivesicular bodies (MVB) containing intraluminal vesicles (ILV). The ILV are released as exosomes when the MVB fuse with the plasma membrane. Microvesicles shed directly from the plasma membrane. EVs facilitate intercellular communication through several processes. They can interact with surface receptors on the recipient cell, or release cargo into the recipient cell either through direct fusion with the cell membrane or by endocytosis.

EVs can be classified into different types according to their size, composition and origin, although a full consensus in terminology is yet to be reached in the research community, and a named category is not necessarily exclusive of vesicles that may belong to other categories [36]. Exosomes, the most widely investigated type of EV, are small vesicles with a size range of 30–150 nm that are derived from the endosomal compartment [37] (Figure 1). Endosomal membrane invagination results in the formation of multivesicular bodies (MVB) containing intraluminal vesicles (ILV), which selectively encapsulate certain nucleic acids, proteins and lipids. Once the MVB fuses with the plasma membrane, the ILV are released directly into the extracellular space as exosomes. Due to their endosomal origin, exosomes are characterised by the expression of endosomal markers, including tetraspanins (CD9, CD63, CD81 and CD82), Flotillin-1 and -2, as well as Alix and TSG101 from the endosomal sorting complex required for transport (ESCRT). Microvesicles, previously known as microparticles, have some size overlap with exosomes, but can be much larger, ranging between 50–1000 nm [38] (Figure 1). They shed directly from the plasma membrane, and as such express membrane markers from the parental cells. Similar to exosomes, microvesicles may contain nucleic acids, proteins and lipids, and can transport these signalling molecules to target cells. Apoptotic bodies are the largest type of EV, exceeding 1000 nm in diameter, and are membrane blebs formed during the late stage of cell apoptosis [38]. They are of interest in biomarker research, but there is no evidence on their role in intercellular communication or usefulness in regenerative medicine.

EVs can communicate with recipient cells through different mechanisms [38,39] (Figure 1). They can interact with cell surface receptors through their transmembrane proteins, thereby inducing intracellular signalling pathways. They can release cargo into target cells, either through direct fusion with the cell membrane or by endocytosis. This can result in dramatic changes in the recipient cells, as shown notably in the field of cancer [40]. The cargo and function of EVs depend on their cells of origin and the conditions under which the EVs were produced. For example, cellular stress is known to modulate EV content and downstream intercellular communications [41]. By gaining a better understanding of the biological functions of EVs and manipulating their biogenesis, research methods in regenerative medicine aim to control their pathophysiological effects in disease development, and produce populations of EVs with protective and pro-regenerative effects.

### 4.2. Advantages of Using Extracellular Vesicles for Regenerative Medicine Applications

Using EVs has numerous advantages over cell therapy for regenerative medicine applications. EVs can bypass most of the safety concerns associated with direct cell transplantation, such as the risk of emboli formation due to intravenous infusion, pathological transformation or tumorigenesis due to genetic abnormalities or uncontrolled cell differentiation, or immune activation for allogeneic preparations [1]. Furthermore, unlike transplanted cells which cannot be retrieved, treatment using EVs is not permanent and can be easily stopped in the event of adverse effects. The small size of EVs also opens up potential applications as nanoscale delivery vehicles, without the potential toxicity or immunogenicity associated with artificial carriers such as liposomes or nanoparticles [42]. From a practical perspective, the production of EVs is more amenable to process optimisation and clinical upscaling to ensure reproducibility and cost-effectiveness, such as by allowing the controlled selection of cell sources and the possibility of adopting cell lines with infinite expansion potential. Unlike cells, the generated EVs can be evaluated for safety, dosage and potency using similar methods as for conventional pharmaceutical agents, which can greatly accelerate the route to clinical translation. Furthermore, purified EVs can be stored for long periods of time without loss of biological activity (−20 °C for 6 months [43] or −80 °C for up to 2 years [44]). This circumvents common storage and transport issues and facilitates the use of EVs as ‘off-the-shelf’ therapeutic agents.

Using MSC-derived EVs for treating joint injury and osteoarthritis presents several distinct advantages. Firstly, MSC-derived EVs possess the same immune-privileged properties as MSCs, which permits allogeneic therapy without the inherent risk of antigen presentation in MSCs following differentiation into more specialised cell types. Secondly, signals carried in MSC-derived EVs are more stable, as they are ‘locked’ at a specific point in the growth or differentiation of the origin MSCs under controlled conditions, which removes concerns regarding the long-term biological behaviour of MSCs, such as senescence following prolonged cell expansion, or cartilage calcification following chondrogenic induction. Last but not least, the removal of ‘environmentally-responsive’ properties in MSC-derived EVs compared with MSCs is advantageous for use in a pathological joint environment, as the EVs will not be prone to adopting the inflammatory profile of the resident joint tissues and cells.

### 4.3. Effects of Extracellular Vesicles in Regenerative Medicine Applications

**Figure 2:**
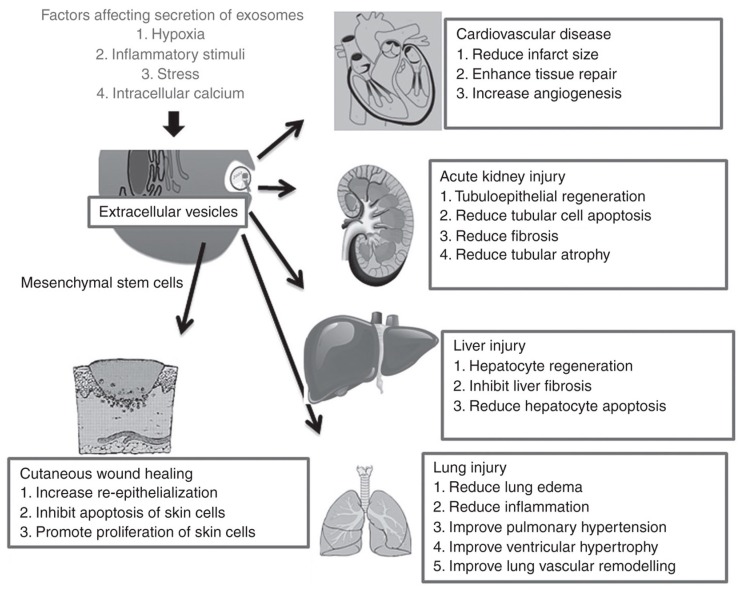
Effects of extracellular vesicles in a variety of regenerative medicine applications [35]. MSC-derived EVs in particular have shown therapeutic benefits by promoting repair and regeneration in numerous tissue types, including the heart, kidney, liver, lung and skin. Reproduced with permission from [35].

EVs derived from specific cell types and under specific conditions have been shown to promote regeneration in a wide range of tissues, including the heart and blood vessels, kidney, liver, lung, skin, neural tissue, and reproductive tissue [1,34,35] (Figure 2). MSCs are the most widely used source cells for generating EVs in these applications, and it is thought that the MSC-derived EVs share the same anti-inflammatory and trophic properties as the parental MSCs to exert their therapeutic effects. For instance, MSC-derived EVs have been shown to have positive effects on cell viability and proliferation [45,46,47], angiogenesis [48,49,50], and immunomodulation [51,52,53] in a range of physiological systems. Although not as widely explored as for other tissue types, a handful of studies have attempted to harness the same paracrine effects of EVs, in particular, MSC-derived EVs, for improving treatment outcomes in experimental models of joint injury and osteoarthritis.

### 4.4. Role of Extracellular Vesicles in The Pathogenesis and Progression of Osteoarthritis

**Figure 3:**
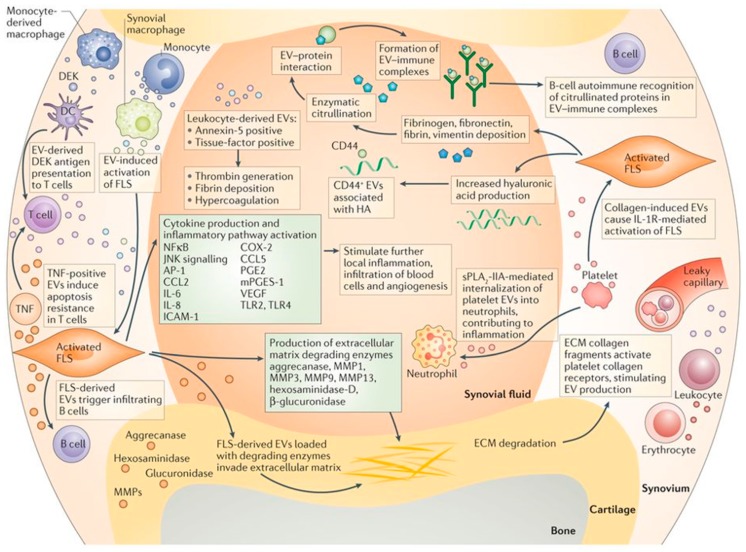
Proposed mechanisms for EV-mediated communication in joint inflammation and disease pathogenesis [2]. EVs are thought to be responsible for intercellular communication among immune cells, fibroblast-like synoviocytes (FLS), chondrocytes and bone cells within a diseased joint environment, leading to chronic inflammation, matrix degradation and irreversible progression of joint degeneration. Reproduced with permission from [2]. Copyright Springer Nature, 2016.

The pathogenesis of osteoarthritis is complex and incompletely understood. However, ample evidence suggests that the process is orchestrated by intricate cross-talk among resident and circulating cells (synoviocytes, chondrocytes, bone cells, immune cells), the extracellular matrix of numerous tissues (synovium, articular cartilage, meniscus, ligament, subchondral bone), and biological fluids (synovial fluid) within the joint environment [3,54]. EVs secreted by different cell types within the joint are thought to facilitate these communications, thereby mediating both healthy joint homeostasis and the pathogenesis and progression of osteoarthritis [55,56]. For instance, one set of proposed mechanisms for joint inflammation and disease pathogenesis is that infiltrating leukocytes and resident synovial macrophages activate fibroblast-like synoviocytes in the synovial membrane through EV-mediated intercellular communication [2] (Figure 3). The activated synoviocytes further maintain joint inflammation through the production of cytokines and enzymes, while releasing EVs that send inflammatory signals back to the immune cells. These EVs can also invade the cartilage extracellular matrix and discharge enzymes, leading to matrix degradation and subchondral bone changes. These interlinked processes form a feedback loop, converting the joint into a catabolic environment that catalyses joint degeneration and irreversible progression of osteoarthritis.

So far, only one published study has specifically investigated the role of EVs in transmitting pathogenic signals between cell types relevant in osteoarthritis, through a set of in vitro experiments [57] (described later in Table 1), and more research is warranted in this direction. Excitingly, a first report that synovial fluid microRNA content is altered in patients with osteoarthritis, and that these changes are gender-specific, suggests the prospect of using EVs to detect tissue-specific changes as biomarkers for osteoarthritis [58]. For this to be successful, future studies will need to investigate the detection of joint-specific EVs in the circulation, as well as the accurate identification of the cellular origin of these EVs.

### 4.5. Current Evidence on The Effects of EVs in Joint Injury and Osteoarthritis

The currently available evidence on the effects of EVs in joint injury and osteoarthritis can be divided into several categories (Table 1): (1) the effects of EVs in experimental models of osteoarthritis [59,60,61,62,63,64], (2) the role of EVs in the pathophysiology of osteoarthritis [57], (3) the effects of EVs in experimental models of inflammation [65,66,67], and (4) the effects of EVs on the regeneration of cartilage or osteochondral tissue [68,69,70]. Within this relatively small number of studies, the findings in relation to the effects of EVs in the model systems tested have been fairly consistent. The majority of studies used MSCs as source cells to generate the EVs, which may be derived from adult tissues (bone marrow [59,60,62,65], adipose tissue [67], synovial membrane [61,64]) or pluripotent cells (embryonic stem cells (ESCs) [63,69,70], induced pluripotent stem cells (iPSCs) [64,68]). Most studies exclusively tested exosomes [57,60,61,63,64,68,69,70], while others compared the effects of exosomes and microvesicles from the same cell source [59,65], and the remaining studies tested a more heterogeneous EV population that likely contained both exosomes and microvesicles [62,66,67]. The terms used to describe sub-categories of EVs were defined differently across studies, with exosomes typically referring to smaller particles below 150 nm, while microvesicles and microparticles were used interchangeably to describe larger particles.

**Figure 4:**
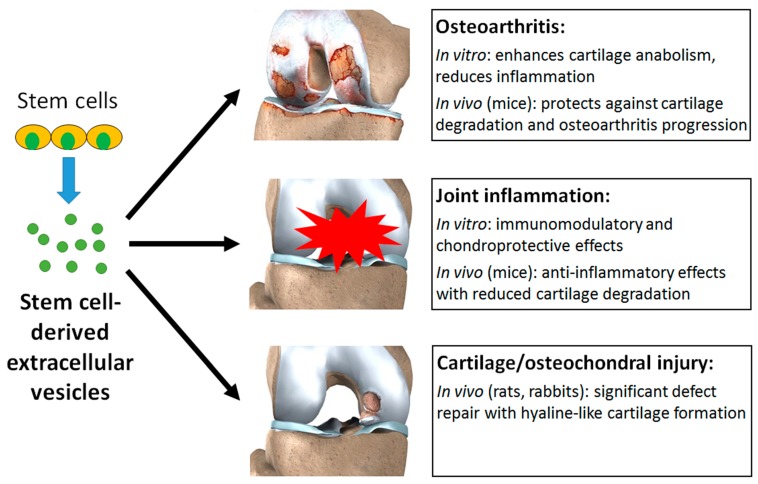
Stem cell-derived EVs can exert a multitude of beneficial effects in experimental models of osteoarthritis, joint inflammation, and cartilage or osteochondral injury.

Stem cell-derived EVs were shown to have a multitude of beneficial effects in experimental models of osteoarthritis, inflammation, and cartilage injury (Figure 4). In osteoarthritis models, EVs enhanced cartilage anabolism and reduced inflammation in vitro [59,60,62,63], and had protective effects against cartilage degradation and osteoarthritis progression in mice following joint injury [59,60,61,63,64]. In models of joint inflammation, EVs had immunomodulatory and chondroprotective effects in vitro, with the ability to influence the behaviour of chondrocytes [66] and multiple types of immune cells [65,67], which were translated to prominent anti-inflammatory effects in vivo (mice) [65,66,67]. The regenerative effects of EVs were demonstrated in osteochondral defects created in rat [69,70] and rabbit [68] models, all of which resulted in significant defect repair with formation of hyaline-like cartilage abundant in collagen type II. These effects were attributed to the creation of a regenerative immune phenotype and elevation of chondrocyte metabolic activity within the EV-treated defects [70]. Embedding EVs in a hydrogel in situ was found to significantly improve cartilage repair compared to EV injection alone, possibly due to better localisation or concentration of the EVs within the defect [68].

### 4.6. Limitations in Translating Stem Cell-Derived Extracellular Vesicles As Therapeutic Agents for Treating Joint Injury and Osteoarthritis

Despite encouraging first results in small animals, the therapeutic efficacy of stem cell-derived EVs needs to be confirmed in larger animals, such as horses and sheep, before development into a clinical therapy can be considered for the treatment of joint injury and osteoarthritis. Proof of efficacy studies also need to be performed over longer time periods, exceeding the currently defined end points at which cartilage repair or attenuation of osteoarthritis progression are typically evaluated in animal models. This is to not only to ensure that EVs delivered to the joint exert initial anti-inflammatory and pro-regenerative effects, but also to confirm that these effects are maintained over time, such that any regenerated cartilage is preserved and halting of disease progression is permanent, rather than eventual reversion back to a pathogenic phenotype. It is possible that the repeated administration of EVs will be necessary to maintain long-term effects, and further studies will be necessary to determine the appropriate timing, dosage and frequency of EV administration, which may be partly dependent on the severity of disease and the type of joint affected. The immobilisation of EVs using a suitable biomaterial, such as encapsulation within a hydrogel or binding onto the surface of a scaffold, may be beneficial for the sustained delivery and improved localisation of EVs within the treated joint, and may reduce the necessary dosage and frequency of administration.

Due to their short history of applications in joint disease, many unanswered questions remain regarding the observed therapeutic effects of stem cell-derived EVs in vitro and in small animal models. EVs produced by MSCs derived from different types of body tissues or pluripotent cells vary in their cargo and therefore effects on target cells [64]. The appropriate selection of source cells and their stage of differentiation is likely to be a critical factor in producing EVs with optimal characteristics to suit specific applications, such as reducing inflammation, enhancing cartilage regeneration, or protecting against degeneration in the joint. However, only one study so far has compared the effects of using different types of source cells for generating EVs [64], and the selection process is far from standardised. Furthermore, although a multitude of beneficial effects have been observed when applying EVs in experimental models of joint inflammation, cartilage damage and osteoarthritis, the mechanisms underlying these effects are not well understood. The signalling pathways involved were either not investigated or only investigated in selected cell types rather than considering the whole joint. Although this is a complex issue to address, the information is essential in understanding the specific role of EVs in facilitating intercellular communication within the diseased joint and producing a targeted therapeutic response. Following on from this, the exact contributions of different sub-categories of EVs, namely exosomes and microvesicles, in promoting joint repair or slowing degeneration need to be better elucidated and compared.

From a practical perspective, a standardised set of techniques needs to be developed for the reproducible generation, purification and characterisation of stem cell-derived EVs for clinical upscaling. Current methods for EV isolation include centrifugation, size exclusion, immunoaffinity isolation, polymeric precipitation, and microfluidic devices [71]. However, all of these methods have different limitations such as the inability to exclude certain contaminating materials, possible loss of EV function following isolation, failure to completely isolate EV fractions, and low yield [72]. The outcome of EV isolation is highly dependent on the cell source and conditions under which the cells are cultured [73]. For instance, EVs derived from immortalised cell lines typically have higher yields, but their function is less well characterised, and may carry some risk of oncogenic potential. On the other hand, EVs derived from primary cells (including MSCs) have well-characterised functions, but may also have lower yields, and their generation can be limited by the finite expansion potential of the source cells. The type of media and substrate used to culture the cells can also independently affect the EV composition. Diverse methods are used for EV characterisation, such as quantification through nanoparticle tracking analysis or dynamic light scattering, morphological analysis through transmission electron microscopy, and protein analysis through Western blotting or flow cytometry [74]. For industrial scale production of EVs to be possible for therapeutic applications, standardised methods need to be established to ensure the homogeneity of EV preparations, as well as their safety and efficacy for downstream applications. The International Society for Extracellular Vesicles (ISEV) has released position papers and the Minimal Information for Studies on EVs (MISEV) to help overcome some of these problems [72].

A number of factors specifically relating to the application of stem cell-derived EVs as therapeutic tools for treating joint injury and osteoarthritis should be considered. Challenges are often experienced in generating sufficient quantities of EVs for in vivo animal studies or human clinical trials. For example, 1 L of MSC-conditioned media from a total of approximately 60 million MSCs yields 1–2 mg (protein content) EVs in an experimental setting [75]. While this yield may be insufficient to produce a therapeutic response by systemic administration, it may be suitable for localised application in the joint by intra-articular injection, considering that previous human trials testing intra-articular cellular therapy for osteoarthritis and focal cartilage defects of the knee generally injected cells in the range of 1–20 million [18]. To ensure optimal localisation and efficacy of administered EVs within the joint, appropriate methods for the storage and recovery of EVs should be developed to maintain their biological potency for off-the-shelf use [76], as well as suitable delivery vehicles to facilitate the controlled and sequential release of EVs into the intra-articular space [2]. Engineering advances in biomaterials and bioprinting technologies may be able to assist the development of multiphasic constructs containing different populations of EVs, to produce a spatially and temporally controlled set of orchestrated responses in the joint environment.

## 5. Summary and Outlook

The use of stem cell-derived EVs for the treatment of joint injury and osteoarthritis is a new and exciting area of investigation in regenerative medicine. So far, the available evidence based on a relatively small number of studies has indicated that EVs, particularly those derived from MSCs, can exert a therapeutic effect in a diseased joint environment by increasing cell viability and proliferation, suppressing inflammation, inducing tissue repair and regeneration, and offering protection against osteoarthritis progression. EVs can theoretically deliver the same trophic signals as their parental cells, but provide a much simpler, safer, more practical and more easily controlled solution compared to direct cell transplantation. The implementation of EV-based products for clinical testing is expected to be accelerated, despite their relatively short history of discovery and experimentation, due to the safety data already available for a range of cell types from which EVs can be derived. For example, MSCs have already been tested and generally found to be safe in over 600 clinical trials [76]. More studies are now needed to better elucidate the therapeutic effects and mechanisms, kinetics, and biodistribution of EVs in the joint environment in relation to injury and osteoarthritis, as well as to decipher the influence of cell sources and conditions for EV generation, different EV sub-categories, dosage and frequency of EV administration, and the type of target cell or joint. Future EV-based products, which are the facilitators of intercellular communication, will benefit from interdisciplinary communication between biologists and engineers to produce a combined therapeutic approach, which integrates fundamental knowledge on the physiological and pathophysiological functions of EVs with enabling technologies to optimise their manufacturing and delivery. Although many research questions remain to be answered, stem cell-derived EVs hold significant promise for use as a new therapeutic tool to provide a curative solution in the treatment of joint injury and osteoarthritis, and alleviate the global burden associated with these debilitating conditions.

## Figures and Tables

**Table 1:** Current evidence on the effects of EVs in joint injury and osteoarthritis. Breg = regulatory B-cell; DMM = destabilisation of the medial meniscus; ECM = extracellular matrix; ESC = embryonic stem cell; HUVEC = human umbilical vein endothelial cells; ICRS = International Cartilage Repair Society; IFN = interferon; IL = interleukin; iPSC = induced pluripotent stem cell; miRNA = microRNA; MMP = matrix metalloproteinase; MSC = mesenchymal stem cell; MV = microvesicle; PBS = phosphate buffered saline; PGE2 = prostaglandin E2; OA = osteoarthritis; OARSI = Osteoarthritis Research Society International; RA = rheumatoid arthritis; TGF = transforming growth factor; TNF = tumor necrosis factor; Tr1 = type I regulatory T-cell; Treg = regulatory T-cell; VEGF = vascular endothelial growth factor.

**(1) Effects of EVs in experimental models of osteoarthritis**				
**Study**	**Source of EV**	**EV type (s)**	**Model(s) for testing EV**	**Findings**
(**1**) Cosenza 2017 [59]	Murine primary bone marrow-derived MSCs	Exosomes (<120 nm; expressed CD9, CD81) and microparticles (~400 nm; expressed CD29, CD44, Sca-1)	- Incubated with OA-like murine chondrocytes for 24 h - Incubated with murine spleen-derived macrophages for 72 h - Injected in knee joint of collagenase-induced OA mouse model at day 7 after OA induction; harvest at day 42	- In vitro, both exosomes and microparticles enhanced the expression of anabolic cartilage markers (collagen type II, aggrecan) in OA-like chondrocytes in a dose-dependent manner, and inhibited catabolic (MMP-13, ADAMTS5) and inflammatory (iNOS) markers; both protected chondrocytes from induced apoptosis (exosomes were more efficient than microparticles) and inhibited macrophage activation. - In vivo, MSCs, exosomes and microparticles had equal effects in protecting treated mice from joint damage, achieving similar values for cartilage and subchondral bone parameters (volume, degradation) as healthy mice; all reduced calcification of ligaments and menisci. - Exosomes and microparticles had similar chondroprotective and anti-inflammatory functions in vitro, and both protected mice from developing OA in vivo, reproducing the effects of MSCs.
(**1**) Mao 2018 [60]	Human bone marrow-derived MSCs, native or transfected (with miR-92a-3p mimic or inhibitor)	Exosomes (50–150 nm; expressed CD9, CD63, CD81, HSP70)	- Incubated with human MSCs undergoing chondrogenesis, and normal and OA primary human chondrocytes - Injected into collagenase-induced OA mouse model at days 7, 14 and 21 after OA induction; harvest at day 28	- Expression of miR-92a-3p in exosomes was significantly upregulated after chondrogenic differentiation of MSCs, but was significantly reduced in exosomes secreted by OA chondrocytes compared to normal cartilage. - OA chondrocytes cultured with exosomes showed greater proliferation and motility, with MSC-miR-92a-3p exosomes having a greater effect than native MSC exosomes. - MSC-miR-92a-3p exosomes upregulated chondrogenic markers (including aggrecan, COL2A1, SOX9) and downregulated other (such as matrix degradation-related) markers (including COL10A1, RUNX2, MMP-13, WNT5A) in MSCs during chondrogenesis and in OA chondrocytes, suggesting effects in enhancing cartilage development and slowing OA progression; MSC-anti-miR-92a-3p had opposite effects. - The above effects were due to miR-92a-3p inhibiting WNT5A expression in both MSCs and OA chondrocytes. - Native MSC and MSC-miR-92a-3p exosomes both significantly inhibited cartilage degradation in mice, with MSC-miR-92a-3p exosomes having a greater effect and almost matching the composition of normal tissues.
(**1**) Tao 2017 [61]	Human synovial membrane-derived MSCs, transfected or not with miR-140-5p	Exosomes (30–150 nm; expressed CD9, CD63, CD81, Alix)	- Incubated with human articular chondrocytes from knee joint for 24 h - Injected into OA rat model (surgically-induced knee instability) weekly in the 5th-8th week after surgery; harvest at 12 weeks	- In vitro, exosomes from native synovial MSCs enhanced chondrocyte proliferation and migration, but with the side effect of significantly reduced ECM secretion; these effects were due to Wnt5a and Wnt5b carried by exosomes, which activated YAP through the alternative Wnt signalling pathway. - Exosomes from synovial MSCs overexpressing miR-140-5p enhanced chondrocyte proliferation and migration without obvious reduction of ECM secretion; this was due to miR-140-5p suppressing RalA and upregulating SOX9, ACAN and collagen type II. - In vivo, MSC-miR-140-5p exosomes slowed progression of early OA and prevented severe damage to knee articular cartilage, with a thick layer of collagen type II-abundant cartilage matrix, no decrease in aggrecan expression and no collagen type I expression; native MSC exosomes were inferior to MSC-miR-140-5p exosomes but superior to the OA control group, with thin cartilage matrix, low aggrecan expression and obvious collagen type I expression.
(**1**) Vonk 2018 [62]	Human bone marrow-derived MSCs	EV (containing exosomes 40–150 nm, expressing CD9 and CD63, and larger particles >150 nm)	- Incubated with human knee OA chondrocytes, pre-treated with TNF-α for some experiments	- EVs were rapidly taken up by OA chondrocytes (after 30 min incubation). - EVs had anti-inflammatory effects when incubated with TNF-α stimulated OA chondrocytes for 48 h, mediated by inhibition of the NFκB signalling pathway; EVs downregulated TNF-α mediated COX2 expression in OA chondrocytes, and expression of pro-inflammatory interleukins (IL-1α, IL-1β, IL-6, IL-8, IL-17); EVs inhibited TNF-α induced collagenase activity in OA chondrocytes, and increased their proliferation. - OA chondrocytes treated with EVs every 5 days for 28 days showed improved in vitro cartilage regeneration; EVs significantly increased proteoglycan and collagen type II content in the newly formed tissue, and expression of ACAN and COL2A1; EVs increased expression of SOX9 and WNT7a, and downregulated the hypertrophy markers RUNX2, COL10A1 and ALP; EVs increased metabolic activity of OA chondrocytes during cartilage regeneration (proliferation, DNA content).
(**1**) Wang 2017 [63]	Human male H1 ESC-derived MSCs	Exosomes (30–200 nm; expressed CD9, CD63)	- Incubated with murine primary articular chondrocytes, treated or not with IL-1β - Intra-articular injection into destabilisation of the medial meniscus (DMM) OA mouse model, every 3 days from week 4 after DMM surgery; harvest at 8 weeks	- Intra-articular injection of ESC-derived MSCs directly into the DMM model alleviated cartilage destruction and matrix degradation, which was mediated by exosomes. - In vitro, IL-1β treatment in chondrocytes inhibited collagen type II synthesis and increased ADAMTS5 expression, which were reversed by conditioned medium from ESC-derived MSCs but not by exosome-depleted conditioned medium; isolated exosomes added to IL-1β treated chondrocytes had the same effect as conditioned medium (increased collagen type II expression and reduced ADAMTS5 expression); added exosomes were detected within collagen type II-expression chondrocytes. - In vivo, exosomes alleviated cartilage destruction in the DMM model and resulted in milder pathology, with the cartilage displaying increased amount of collagen type II and aggrecan, and reduced ADAMTS5. - Exosomes from ESC-derived MSCs may have a therapeutic effect on OA by balancing the synthesis and degradation of chondrocyte ECM.
(**1**) Zhu 2017 [64]	Human iPSC-derived MSCs, and synovial membrane MSCs	Exosomes (50–150 nm; expressed CD9, CD63, TSG101)	- Incubated with human articular chondrocytes - Intra-articular injection into collagenase-induced OA mouse model at days 7, 14 and 21 after OA induction (normal group = saline instead of collagenase); harvest at day 28	- In vitro, both iPSC-MSC and synovial MSC exosomes enhanced chondrocyte migration and proliferation, with the iPSC-MSC-derived exosomes having a greater effect. - In vivo, both iPSC-MSC and synovial MSC exosomes attenuated OA in the mouse model, but the iPSC-MSC exosomes had a much stronger therapeutic effect; ICRS macroscopic assessment scores of the normal and two exosome groups were similar and significantly higher than the OA group; iPSC-MSC exosomes achieved the best histological repair (smooth cartilage surface, regular cellular organisation, normal proteoglycan content) and lowest OARSI score (similar to normal group), while the OARSI score for synovial MSC exosomes was significantly higher than for iPSC-MSC exosomes but was still significantly lower than the OA group; collagen type II staining was more intense in the normal and two exosome groups than the OA group (superficial and deep zones for iPSC-MSC exosomes, but only weakly in the superficial zone for synovial MSC exosomes), while collagen type I staining was only present in the OA group.
**(2) Role of EVs in the pathophysiology of osteoarthritis**				
**Study**	**Source of EV**	**EV type (s)**	**Model(s) for testing EV**	**Findings**
(**2**) Kato 2014 [57]	Human synovial fibroblasts from normal knee joint (stimulated or not with IL-1β)	Exosomes (60–200 nm; expressed CD9, CD81, flotillin-1)	- Incubated with human articular chondrocytes from normal knee joint for 24 h - Incubated with mouse femoral head cartilage explants for 72 h - Incubated with human umbilical vein endothelial cells (HUVECs) for 4 h	- Inflammatory cytokines (IL-6) and angiogenic factors (VEGF, MMP-3) were increased in conditioned medium and exosomes from IL-1β stimulated fibroblasts, and were present mostly in the conditioned medium with low levels in exosomes; IL-1β, TNF-α, MMP-9 and MMP-13 were not detectable in conditioned medium or exosomes from fibroblasts. - Exosomes from IL-1β stimulated fibroblasts induced OA-related gene expression in chondrocytes, including significant upregulation of MMP-13 and ADAMTS-5, and downregulation of COL2A1 and ACAN. - Exosomes from IL-1β stimulated fibroblasts significantly increased proteoglycan release from mouse cartilage explants compared to those from non-stimulated fibroblasts. - Exosomes from IL-1β stimulated fibroblasts contained angiogenic signals, and significantly increased migration and tube formation activity in HUVECs, while direct IL-1β application had no effect. - Levels of 50 miRNAs were differentially expressed in exosomes from IL-1β stimulated fibroblasts compared to non-stimulated fibroblasts (NanoString analysis). - Exosomes may be a mechanism by which pathogenic signals are communicated between cell types in OA-affected joints.
**(3) Effects of EVs in experimental models of inflammation**				
**Study**	**Source of EV**	**EV type (s)**	**Model(s) for testing EV**	**Findings**
(**3**) Cosenza 2018 [65]	Murine bone marrow-derived MSCs	Total EV, separated into exosomes (~120 nm; expressed CD9, CD81) and microparticles (150–600 nm; expressed CD29, CD44, Sca-1)	- Incubated with murine T and B lymphocytes for 3 days - Injected into the footpad in delayed T hypersensitivity mouse model 5 days after immunisation; harvest at day 6 - Injected intravenously in collagen-induced arthritis mouse model at days 18 and 24; harvest at day 30	- Immunomodulatory activity of EVs was lost after freeze-thawing; analyses were performed using freshly prepared EVs kept at 4 °C for less than 24 h. - Exosomes and microparticles indirectly inhibited T lymphocyte proliferation in a dose-dependent manner (through Tr1 and Treg induction), and decreased the percentage of CD4+ and CD8+ T-cell subsets; exosomes and microparticles increased Treg cell populations while parental MSCs did not. - MSCs, exosomes and microparticles had similar effects in reducing plasmablast differentiation. - IFN-γ priming of MSCs before EV isolation did not influence the immunomodulatory function of isolated exosomes or microparticles. - In delayed T hypersensitivity model, exosomes and microparticles had a dose-dependent anti-inflammatory effect. - In collagen-induced arthritis model, total EVs and exosomes suppressed clinical signs of inflammation; exosomes were more efficient than microparticles in protecting mice from developing arthritis; beneficial effect of exosomes was associated with fewer plasmablasts and more Breg-like cells in lymph nodes.
(**3**) Headland 2015 [66]	Human RA synovial fluid; human neutrophils (stimulated or not with TNF-α)	Microvesicles (0.05–1 μm) containing exosomes and microparticles; expressed CD66b, annexin V, phalloidin, MRP8, MRP14, annexin A1 (AnxA1)	- Incubated with human chondrocyte micromasses (from C28/I2 cell line or primary articular chondrocytes) - Co-cultured with ex vivo rat cartilage explants for 18 h - Intra-articular injection into inflammatory arthritis mouse models (K/BxN – injected day 3, harvested day 5; glucose-6-phosphate isomerase (G6PI)-induced–injected day 21, harvested day 25)	- In vitro, neutrophil-derived MVs led to cartilage protection in human chondrocytes (reduced chondrocyte apoptosis, IL-8 and PGE2 release, and ECM degradation), through TGF-β induction followed by upregulation of genes key to cartilage anabolism. - Neutrophil-derived (but not macrophage-derived) MVs could actively migrate into rat cartilage explants, and intact MV structures were required for chondroprotection; neutrophils showed increased migration into inflamed joints to release MVs locally; MV contact with human chondrocytes yielded chondroprotection, but direct neutrophil contact with chondrocytes inhibited anabolism and induced cell death. - In vivo, neutrophil-derived MVs reduced cartilage degradation and proteoglycan loss in mouse models of inflammatory arthritis. - Cartilage protection by MVs requires AnxA1 (higher content in MVs produced by TNF-α stimulated neutrophils) and interactions with its receptor FPR2/ALX, which increase TGF-β production by chondrocytes.
(**3**) Lo Sicco 2017 [67]	Human adipose tissue-derived MSCs, cultured in normoxic (20% O_2_) or hypoxic (1% O_2_) conditions	EV (40–250 nm, expressed CD81 and Alix; mostly but not limited to exosomes)	- Subcutaneous injection into mouse angiogenesis model; harvest at 3 weeks - Incubated with murine bone marrow-derived macrophages - Intramuscular injection into mouse model of cardiotoxin-induced muscle injury (2 h and 4 days after injury); harvest at days 1, 2 and 7	- Both types of EV induced angiogenesis, but EVs secreted under hypoxic conditioning of MSCs induced higher expression of angiogenic factors and vessel density, and differentially expressed a number of miRNAs actively involved in wound healing. - Both types of EV were efficiently internalised by macrophages and significantly increased their proliferation, with hypoxic EV having a greater effect than normoxic EV; both induced a significant switch of recipient macrophages to an anti-inflammatory phenotype (M1 to M2) after treatment for 72 h, with hypoxic EVs showing a greater anti-inflammatory effect. - In the skeletal muscle injury model, both types of EV significantly mitigated the inflammatory milieu in the injured tissue at day 1 (reduced IL-6/IL-10 ratio), accompanied by significant increase in M2 markers and decrease in M1 markers at day 2; both resulted in accelerated muscle regeneration (increased expression of myogenic markers and fibre repair) at day 7; hypoxic EVs had a greater effect than normoxic EVs. - MSC-derived EVs, particularly those obtained under hypoxic conditions, can modulate the inflammatory response following injury and influence downstream regeneration.
**(4) Effects of EVs on the regeneration of cartilage/osteochondral tissue**				
**Study**	**Source of EV**	**EV type (s)**	**Model(s) for testing EV**	**Findings**
(**4**) Liu 2017 [68]	Human iPSC-derived MSCs	Exosomes (50–150 nm; expressed CD9, CD63, CD81)	- Incubated with human bone marrow-derived MSCs and chondrocytes to assess cell migration and proliferation - Implanted in a rabbit full-thickness osteochondral defect (4 mm diameter × 3 mm depth), embedded or not in a photoinduced imine crosslinking hydrogel glue; harvest at day 7	- In vitro, exosomes promoted migration and proliferation of MSCs and chondrocytes; exosomes were effectively retained in the hydrogel, and cells encapsulated in the hydrogel in the presence of exosomes showed increased viability. - In vivo, hydrogel with exosomes (formed in situ) showed seamless integration with surrounding cartilage and bone in the rabbit osteochondral defect; compared to hydrogel alone, hydrogel with exosomes attracted significantly higher cell deposition at 7 days post-implantation, including chondrocytes, inflammation cells, fibroblasts and blood cells. - At 12 weeks post-implantation, hydrogel with exosomes (formed in situ) achieved regeneration of articular cartilage that showed similar macroscopic and histological appearance and structure to native tissue, with abundant collagen type II; the control groups achieved inferior results, with hydrogel alone forming a much thinner cartilage layer, pre-formed hydrogel with exosomes showing poor integration with surrounding tissue, and injected exosomes failing to achieve cartilage repair similar to the empty control. - Embedding exosomes as part of an acellular hydrogel patch can potentially promote cartilage repair while reducing the dosage and frequency of exosome application.
(**4**) Zhang 2016 [69]	Human HuES9 ESC-derived MSCs	Exosomes (modal size of 100 nm; expressed CD81, TSG101, Alix)	- Intra-articular injection into critical-sized osteochondral defects (1.5 mm diameter, 1 mm depth) in the trochlear groove of the distal femur in rats, administered weekly for 12 weeks; harvest at 6 and 12 weeks	- At 6 weeks, exosome-treated defects showed moderate to good neotissue filling, moderate surface regularity and good integration with host cartilage, but the ICRS macroscopic assessment score was not significantly different from PBS-treated control defects. Histologically, 4 of 6 exosome-treated defects showed hyaline cartilage formation with high amounts of glycosaminoglycan and collagen type II, low amount of collagen type I, and complete subchondral bone regeneration, with significantly better histological scores using the modified O’Driscoll system compared to controls. - At 12 weeks, exosome-treated defects showed almost complete neotissue coverage with good surface regularity and complete integration with surrounding cartilage, but the ICRS score was again not significantly different from controls. Histologically, 5 of 6 exosome-treated defects showed smooth hyaline cartilage with matrix staining comparable to native cartilage from age-matched unoperated controls (high glycosaminoglycan and collagen type II, low collagen type I and X), complete subchondral bone regeneration, and good bonding to surrounding tissue, while the remaining sample showed fibrocartilaginous repair; modified O’Driscoll score was significantly better compared to controls, which contained only fibrous tissue.
(**4**) Zhang 2018 [70]	Human E1-MYC 16.3 ESC-derived MSCs	Exosomes (modal size of 100 nm; expressed CD81, TSG101, Alix)	- Incubated with rat primary articular chondrocytes - Intra-articular injection into critical-sized osteochondral defects (1.5 mm diameter, 1 mm depth) in the trochlear groove of the distal femur in rats, administered weekly for 12 weeks; harvest at 2, 6 and 12 weeks	- In vivo results matched the previous study (Zhang 2016); exosome-treated defects showed early osteochondral repair at 2 weeks, with significantly higher areal deposition of collagen type II and significantly lower collagen type I, as well as significantly improved Wakitani score, compared to PBS-treated control defects at all 3 time points. - Exosomes enhanced cellular proliferation and attenuated apoptosis in vivo, as shown through a significant increase in PCNA^+^ cells compared to the control in both the cartilage tissue and overlying synovium at all 3 time points; CCP3^+^ apoptotic cell numbers were significantly lower in exosome-treated defects at 6 weeks. - Exosome-treated defects displayed a regenerative immune phenotype, as shown through significantly higher numbers of M2 macrophages and significantly lower numbers of M1 macrophages than controls at all 3 time points, as well as significantly lower levels of M1-associated pro-inflammatory cytokines (IL-1β, TNF-α) in the synovial fluid at 6 weeks. - In vitro, exosomes incubated with chondrocytes were rapidly endocytosed, reaching peak concentration inside the cells at 12 h; exosomes enhanced chondrocyte migration in a dose-dependent manner; over 72 h, exosomes enhanced chondrocyte metabolic activity and proliferation in a dose-dependent manner, accompanied by significantly increased glycosaminoglycan levels and upregulation of genes associated with chondrocyte survival and proliferation (Survivin, Bcl-2, FGF-2, PCNA), and chondrogenic differentiation (TGF-β1, COMP, COL2A1), as early as 24 h after exposure. - Exosomes modulated chondrocyte functions through exosomal CD73-mediated adenosine activation of AKT and ERK signalling; inhibition of AKT or ERK suppressed exosome-mediated increase in chondrocyte migration and proliferation, but not matrix synthesis.
